# Multiscale environmental drivers of human brucellosis transmission in Xinjiang: A spatiotemporal analysis integrating GAM and MaxEnt modeling (2015–2023)

**DOI:** 10.1371/journal.pntd.0013463

**Published:** 2025-08-26

**Authors:** Peiyao Zhou, Jiangshan Zhao, Di Wu, Feifei Li, Xiaodong Wang, Yanling Zheng, Liping Zhang

**Affiliations:** 1 College of Public Health, Xinjiang Medical University, Urumqi, China; 2 Institute for Parasitic Diseases and Brucellosis Prevention and Control, Xinjiang Uighur Autonomous Region Center for Disease Control and Prevention, Urumqi, China; 3 College of Basic Medical Sciences and Public Health, Jinan University, Guangzhou, China; 4 Institute of Medical Engineering Interdisciplinary Research, College of Medical Engineering and Technology, Xinjiang Medical University, Urumqi, China; Colorado State University, UNITED STATES OF AMERICA

## Abstract

This study investigated the epidemiological characteristics of human brucellosis and the associations with meteorological, environmental, and socio-economic factors in Xinjiang Uygur Autonomous Region (XUAR), China, between 2015 and 2023. Using a Generalized Additive Model (GAM), we analyzed nonlinear associations between meteorological variables and case counts, incorporating optimized lag periods for each climatic factor. The Maximum Entropy (MaxEnt) model was simultaneously applied to evaluate the synergistic effects of environmental and socio-economic determinants on disease distribution patterns throughout the region. Key findings revealed distinct epidemiological patterns, characterized by an initial decline followed by a resurgence in cases, predominantly among males (71.2%) and older age groups. Meteorological analysis identified temperature, precipitation, and wind speed as significant risk factors with time-lagged effects, while higher humidity demonstrated a protective effect. Spatially, population density and vegetation cover were the strongest predictors of disease distribution, with high-risk areas concentrated in central and western XUAR, particularly urban centers such as Urumqi and Kashgar. The models demonstrated strong predictive performance, with MaxEnt achieving an area under the curve (AUC) value of 0.987. These findings highlight the complex interplay of climatic, ecological, and demographic factors in brucellosis transmission. The study recommends enhanced surveillance in high-risk regions, implementation of weather-based early warning systems, and targeted livestock control measures in areas with characteristic environmental risk factors.

## Author summary

Brucellosis is a widespread disease that spreads from animals to humans, particularly in regions with intensive livestock farming, such as Xinjiang, China. While previous studies have focused on the biological aspects of the disease, we still lack a clear understanding of how environmental factors, such as climate and vegetation, influence its spread. In this study, we used advanced statistical models to analyze how weather conditions and land cover affect brucellosis outbreaks over time and space. Our findings reveal that factors like temperature fluctuations and vegetation density have complex, nonlinear effects on disease transmission—initially increasing risk but eventually decreasing it under extreme conditions. By combining two modeling approaches, we were able to predict high-risk areas and identify critical time lags between weather changes and disease outbreaks. This work helps public health officials better anticipate and control brucellosis by understanding the environmental drivers behind its spread.

## Introduction

Human brucellosis is a zoonotic infectious disease caused by bacteria of the genus Brucella [1]. According to reports from the World Health Organization and the Global Health Observatory, the disease has a global distribution with regional variations: in North America, it primarily affects intensive livestock farming regions; in South America, outbreaks are driven by cattle and sheep production; in Europe (particularly Southeast Europe, the Mediterranean, and Southern Europe), the disease burden is highest; while in Asia, high-incidence areas like China and India show strong links to livestock practices and occupational exposure [2]. In China, human brucellosis exhibits distinct geographical and occupational patterns, the highest incidence rates concentrated in the northwest and northeast [3,4]. Vulnerable populations include livestock farmers, laboratory personnel, and veterinarians. Xinjiang’s distinctive geographical environment and climatic conditions have fostered a thriving livestock industry, making it one of China’s most developed pastoral regions. From 2019 to 2022, the incidence rate of brucellosis in Xinjiang ranked third, with an incidence rate of 4.69 per 100,000. This livestock-dominated economic structure, combined with traditional husbandry practices that facilitate close human-animal contact, has created optimal conditions for brucellosis transmission [5]. Consequently, the region has consistently maintained one of the highest brucellosis incidence rates nationwide, establishing itself as a key endemic focus for this zoonotic disease in China [6,7].

Most existing studies have focused on exploring the biological and clinical characteristics of human brucellosis; however, investigations into its epidemiological features—especially regarding climatic influences—remain relatively limited. For the majority of infections, some evidence is available of which environmental factors contribute to the population biology of parasites, vectors and zoonotic hosts [8]. Some research has indicated that brucellosis incidence is significantly correlated with various meteorological factors including temperature, humidity, sunshine duration, wind speed, precipitation levels etc [9]. A study conducted in Aksu region of Xinjiang identified high temperatures combined with low relative humidity and low wind speeds as meteorological factors most strongly associated with increased incidence rates of brucellosis [10]. Another investigation revealed that cases peak around mid-May while demonstrating a significant correlation between brucellosis occurrences and climatic variables exhibiting lags ranging from 1 to 5 months, the maximum correlations observed at different lag periods. The findings imply an intrinsic temporal lag between climatic conditions and the subsequent occurrences of infection.

GAM represent a flexible statistical modeling approach that effectively captures the intricate relationships between explanatory variables and response variables through nonlinear smoothing functions [11,12]. These models are particularly well-suited for analyzing environmental health data characterized by nonlinear dynamics [13]. Conversely, the MaxEnt model is a species distribution prediction framework grounded in niche theory, using limited environmental data to estimate geographical probabilities [14,15]. The MaxEnt model has demonstrated strong performance in assessing spatial risks associated with diseases [16]. Here, we combined these approaches: GAM analyzed time-lagged meteorological effects, while the MaxEnt model integrated environmental and socioeconomic factors for spatial risk mapping. This dual-model framework leverages their complementary strengths—GAM handles nonlinear continuous variables, while the MaxEnt model incorporates discrete and presence-background data—enhancing both predictive accuracy and interpretability of complex environmental interactions.

## Data and methods

### Ethics statement

The study protocol was reviewed and approved by the Xinjiang Uygur Autonomous Region Center for Disease Control and Prevention(Ethics Approval No: 2024-23).

### Study area

The Xinjiang Uygur Autonomous Region, situated in the northwest border of China, spans approximately 1.66 million square kilometers between 73°40 ‘and 96°18’ east longitude and 34°25 ‘and 48°10’ north latitude, making it the largest provincial-level administrative region in China. Its topography follows a distinctive pattern characterized by the juxtaposition of three mountain ranges – Altai, Tianshan, and Kunlun – which enclose the Junggar Basin and Tarim Basin. The Tarim Basin houses the expansive Taklimakan Desert, the largest desert in China. Xinjiang experiences a temperate continental climate marked by ample sunlight, limited precipitation (annual average below 150 mm), significant diurnal temperature fluctuations, and varying precipitation patterns from north to south. Northern Xinjiang exhibits prolonged cold winters and brief cool summers, while southern Xinjiang is relatively warm, arid, and hot during the summer months.The region experiences an annual sunshine duration ranging from 250 to 350 hours, the highest in the country. Diurnal temperature fluctuations often surpass 15°C, with peak temperatures exceeding 20°C. Due to its distance from the sea and mountainous terrain, the area exhibits a dry climate characterized by significant evaporation rates surpassing precipitation levels. Windy conditions are prevalent, particularly in the tuyere zone. These distinctive geographical and climatic features create a favorable natural foundation for the advancement of local animal husbandry.

### Data collection

From 2015 to 2023, data on human brucellosis incidence in the Xinjiang Uygur Autonomous Region were collected from the Center for Disease Control and Prevention of Xinjiang Uygur Autonomous Region. A dataset spanning nine years was organized and refined. Latitude and longitude coordinates of brucellosis distribution points and county administrative centers were acquired to establish a geographic information attribute database for brucellosis cases in the region. Data on TEM (average temperature), PRE (precipitation), WIN (wind speed), and RH (relative humidity) utilized in the GAM model were sourced from the National Qinghai-Tibet Plateau Scientific Data Center (https://data.tpdc.ac.cn). Data on population (POP), gross domestic product (GDP), and Normalized Difference Vegetation Index (NDVI) were sourced from the China Science Source and Environmental Science Data Center (https://www.resdc.cn). Additionally, data on bioclimatic variables (BIO2, BIO7, BIO11, BIO15, BIO17), wind patterns (WIND), solar radiation (SCARD), and vapor pressure (VAPR) were retrieved from the World Climate website (https://worldclim.org/). To mitigate the impact of multicollinearity among environmental variables on the outcomes, we assessed the correlation among these variables using ENMTools software, retaining those with a correlation coefficient > |.8|. Subsequently, 11 variables (GDP, annual normalized vegetation index, population density, vegetation coverage, annual average temperature, annual average relative humidity, annual precipitation, and annual evaporation) were selected from the initial pool of 31environmental variables for inclusion in the MaxEnt model (refer to Table 1). Geographic data were obtained from the National Basic Geographic Information Center (http://www.ngcc.cn/). The map of China referenced in this study is based on the standard map (review number GS (2024) 065).

**Table 1:** The meaning and types of relevant environmental variables in the maximum entropy model.

Variable name	Implication	Resolution ratio	Types of variables
BIO2	Mean diurnal temperature range(°C)	1km	continuous variables
BIO7	Temperature annual range(°C)	1km	continuous variables
BIO11	Mean temperature of coldest quarter(°C)	1km	continuous variables
BIO15	Precipitation seasonality(mm)	1km	continuous variables
BIO17	Precipitation of driest quarter(mm)	1km	continuous variables
POP	Population	1km	continuous variables
GDP	Gross Domestic Product(yuan)	1km	continuous variables
NDVI	Normalized Difference Vegetation Index	1km	continuous variables
WIND	Wind speed(m/s)	1km	continuous variables
SCARD	solar radiation (kJ/m²/day)	1km	continuous variables
VAPR	Vapor Pressure	1km	continuous variables

### Statistical analysis

#### GAM.

In recent years, generalized additive model has been widely used to explore the relationship between meteorological factors and health effects [17]. The main reason is that it can introduce nonparametric smooth function into the model on the basis of generalized linear model, and has unique advantages in explaining the nonlinear relationship between dependent variables and many influencing factors. Each term in GAM model is basically independent, and both linear and nonlinear parameters can be included in the model. Another advantage of generalized additive model is that it can study and explore many kinds of distribution data, such as quasi-Poisson distribution and normal distribution. Its connection function is basically consistent with that of generalized linear model [18]. In this study, GAM model with quasi-Poisson regression connection function was used to estimate the influence of meteorological factors on brucellosis. Previous studies have pointed out that the impact of meteorological on brucellosis has hysteresis. Therefore combining relevant references, the longest lag time of the model is set to 12 months, and the cumulative lag data of meteorological factors and air quality factors are generated from 0-1 months to 0–12 months. To explore the cumulative effect of different lag weeks on brucellosis incidence. The Generalized Cross-Validation (GCV) method is adopted to screen the optimal lag period from the candidate lag orders of 1–12 months. This method achieves the selection of the optimal model by balancing the goodness of fit and complexity of the model. The specific implementation process includes the following key steps: First, for each candidate lag order k (k = 1,2,...) 12), a semi-parametric generalized additive model (GAM) is constructed, in which the meteorological factor and its K-order lag term are processed by penalized spline smoothing, and the model complexity is controlled by restricting its degrees of freedom to be constant at 2. The time trend term also adopts penalized spline fitting, with a fixed degree of freedom of 4 to maintain a stable expression of the long-term trend. After the model is fitted, the GCV statistics corresponding to each lag order are calculated. The lag order that minimizes the GCV statistics is selected as the optimal solution. This result theoretically approximately minimizes the mean square prediction error of the model. The entire calculation process is implemented through the mgcv package of the R language. The specific model is as follows:


$$\;\;\text{log}\left[E(Y_{t})\right]=\beta +{x+s}_{1}(z)+s_{2}(month)$$ (1)

where *Y_t_* is the number of new brucellosis cases per month in *t* months; *β* is the intercept of the entire model; *x* is the meteorological factor or air quality factor to be analyzed, *z* is the confounding factor to be controlled, *month* is the variable used to control the long-term trend, S () is the natural cubic spline function, and the optimal degree of freedom of the spline function is determined by GCV score.

Based on the GAM model, the exposure-response curves between meteorological factors and brucellosis were plotted under the optimal lag time. If the curve is linear or approximately linear, the effect of meteorological factors on brucellosis is estimated directly; otherwise, piecewise linear regression is used.

#### MaxEnt model.

The MaxEnt model is a habitat-based potential species distribution model based on known species and environmental data, overcoming administrative limitations, and can be used to study ecological needs of diseases, predict the risk of disease transmission in specific areas, and assess the percentage contribution and profile of each factor to the results [19,20]. In this study, a predictive model was built based on MaxEnt 3.4.3, randomly selecting 25% of the data as the test dataset and the remaining 75% as the training dataset. During modeling, the environmental variable data set was extracted from the mean data of the interval of case occurrence years, and the coordinates of case data distribution points were all located to the latitude and longitude coordinates of township government. The niche model was established by selecting the maximum iteration times of 5 000, using the knife-cut method, the model repeated running times of 10, setting the output type as logistic, the output file format as ASCII grid, the regulation frequency multiplication as 0.5 [21]. Permutation importance quantifies a feature’s importance by how much shuffling its values worsens model performance.

#### Model performance evaluation.

The area under the receiver operating characteristic (ROC) curve (AUC) in the threshold-independent approach was used to evaluate model quality [22]. The closer the AUC value is to 1, the better the fit between the model and the test data, the higher the sensitivity and accuracy, and the higher the predictive ability of the model [23].

#### Assessment of environmental variables.

Evaluate the relative importance and contribution of each environment variable in the model by knife-cutting (i.e., estimate of the relative contribution of each environment variable to the model, sorted by the absolute value of the function in each iteration of the training data algorithm) [24]. The functional relationship between case occurrence and environmental variables was evaluated by response plots of primary environmental variables. Ranking importance evaluates importance by randomly shuffling the values of a variable and then re-predicting it, calculating the degree to which the accuracy of the model has decreased. It reflects the global impact of the variable on the prediction (including interactions with other variables). The main difference between them is that contribution rate focuses on the independent effect of variables themselves, while ranking importance covers the synergistic effect of variables themselves and other variables [25].

#### Classification of transmission risk regions.

Natural breaks were used to classify the risk levels of the prediction results. The transmission risk levels are no risk area (0 ~ 0.0373), low risk area (0.0374 ~ 0.1421), medium risk area (0.1422 ~ 0.3028), high risk area (0.3029 ~ 0.5234) and extremely high risk area (0.5235-0.9533).

## Results

### Descriptive analysis

**Fig 1:**
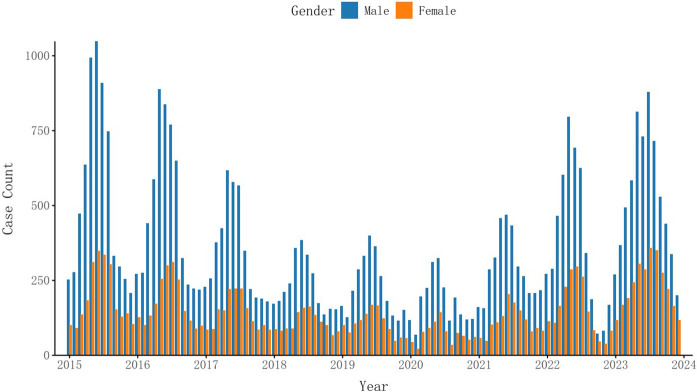
Monthly distribution of incidence of brucellosis between men and women in Xinjiang Uygur autonomous region from 2015 to 2023.

**Fig 2:**
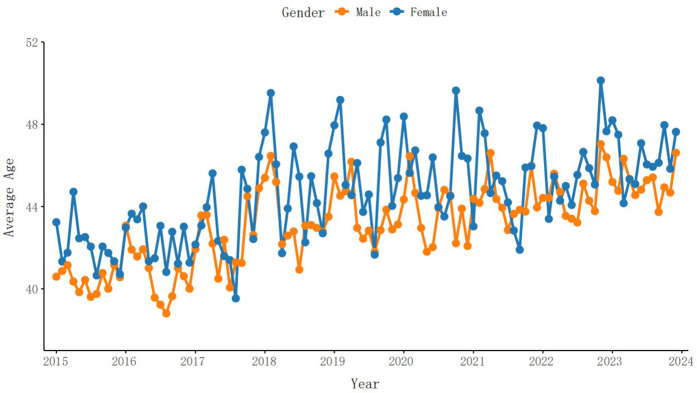
Monthly distribution of average age of brucellosis incidence among men and women in Xinjiang Uygur autonomous region from 2015 to 2023.

**Fig 3:**
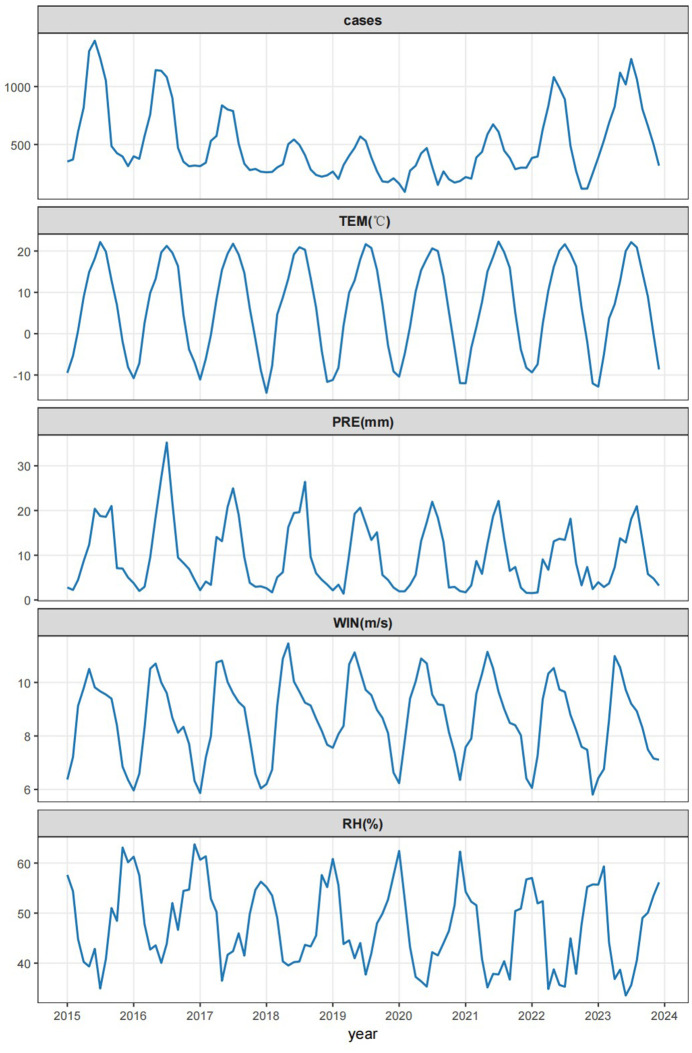
Time series diagram of meteorological factors in Xinjiang Uygur autonomous region from 2015 to 2023.

From 2015 to 2023, a total of 53975 brucellosis cases were reported in Xinjiang Uygur Autonomous Region, including 38433 males and 15542 females, with a minimum of 91 cases per month and a maximum of 1396 cases, with an average of 499.77 cases per month. The number of patients first declined and then rose. Figs 1 and 2 show the monthly incidence of male and female patients and the monthly average age of onset over time. It can be seen that the monthly incidence of male patients and the monthly average age of onset are higher than those of female patients. The number of patients decreases first and then increases, and the age of onset increases. Fig 3 shows the time series curves of monthly meteorological data and monthly number of cases. Mean temperature, relative humidity, wind speed and precipitation all show periodic changes.

### Model performance evaluation

**Fig 4:**
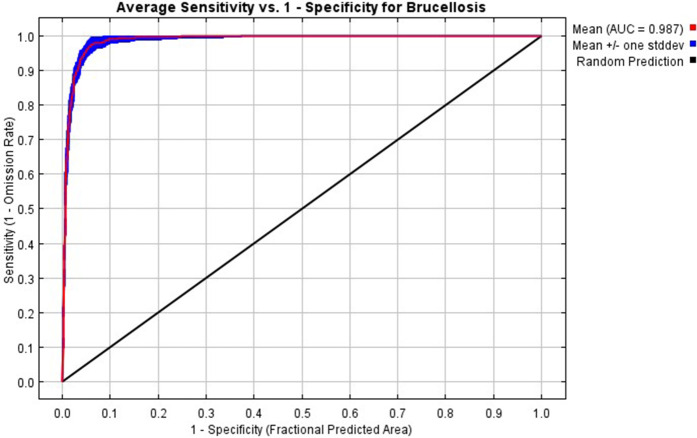
ROC curve of MaxEnt model.

AUC = 0.987 (standard deviation = 0.013) for the model in Fig 4, the model fits well with the test data and has high predictive sensitivity and accuracy.

### Association between meteorological factors and human brucellosis transmission

**Fig 5:**
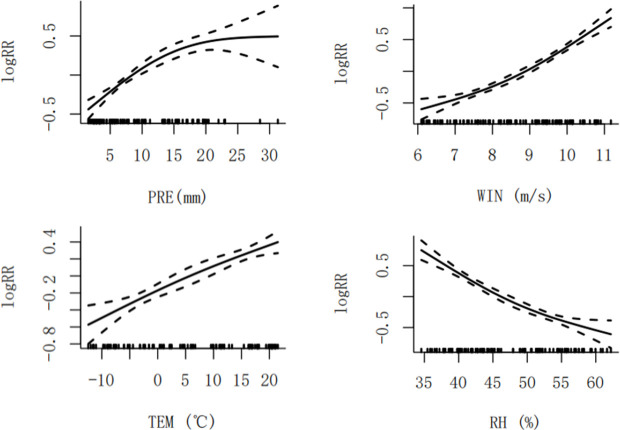
Exposure response curves for average temperature effects on brucellosis.

In this study, generalized additive model was used to analyze the nonlinear relationship between four meteorological factors (mean temperature, precipitation, wind speed and relative humidity) and brucellosis cases. The lag orders of each variable were mean temperature (TEM10, lag 10 order), precipitation (PRE09, lag 9 order), wind speed (WIN01, lag 1 order) and relative humidity (RH01, lag 1 order). Fig 5 shows that high temperature, high precipitation, high wind speed and low relative humidity were risk factors for brucellosis. With the increase of average temperature and wind speed, the risk of brucellosis increased; when monthly precipitation was below 20mm, the risk of brucellosis increased with the increase of precipitation, and when monthly precipitation reached 20mm, the risk of brucellosis tended to be flat; with the increase of relative humidity, the risk of brucellosis decreased.

### MaxEnt model environmental variable importance assessment

**Fig 6:**
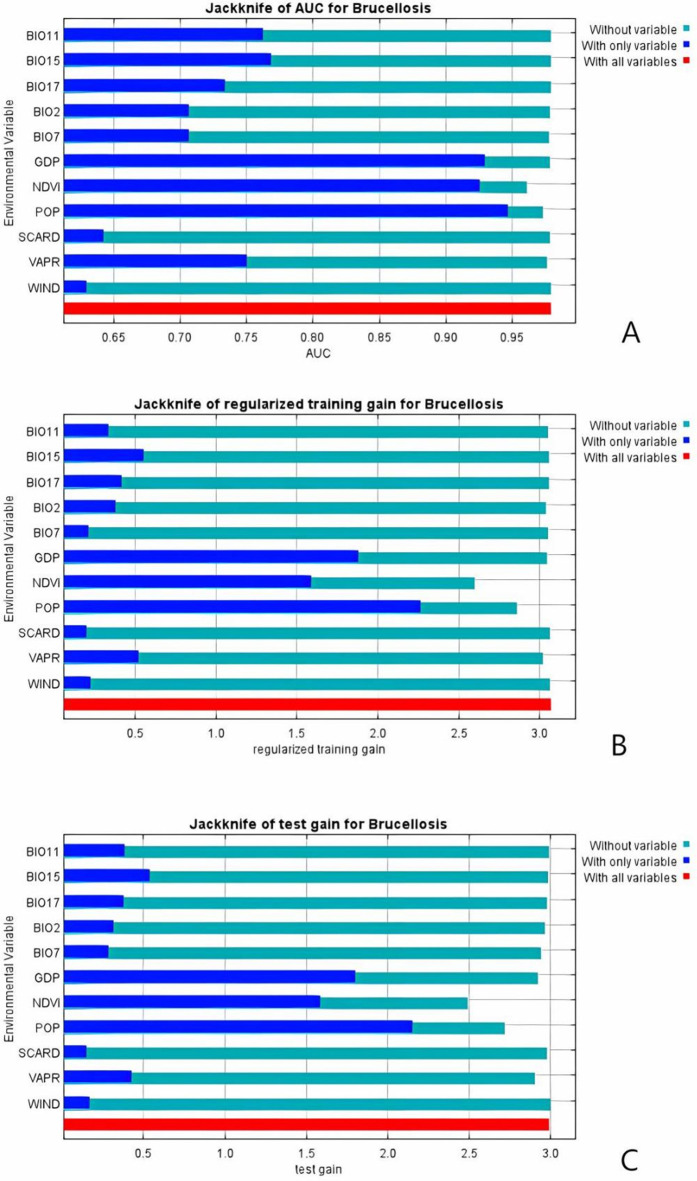
The results of the jackknife test of variable importance. The values presented are the mean values obtained from replicate runs. (A) The jackknife of regularized training gain test. (B) The jackknife of test gain. (C) The jackknife of AUC.

The prediction results of species distribution based on maximum entropy model showed that the contribution rate and permutation importance of different environmental and socio-economic variables to model prediction were significantly different. Population density (POP) showed the highest contribution rate (69.8%) and permutation importance (45.1%) among all variables, and NDVI was the second most important variable, with contribution rate of 20.4% and permutation importance of 30.4%, indicating that vegetation cover had a significant impact on species distribution. In contrast, the contribution to gross domestic product (GDP)(4.4 per cent) and permutation importance (0.6 per cent) are low, indicating limited direct impact on the model. Among climate variables, the coldest seasonal mean temperature (BIO11), although contributing less (0.6%), showed higher permutation importance (7.1%), suggesting that extreme low temperature conditions may be the key factor limiting species distribution. Other bioclimatic variables such as diurnal temperature difference (BIO2), annual temperature range (BIO7) and driest seasonal precipitation (BIO17) all contributed less than 1.5%, but the importance of displacement was moderate (2.6-4.5%), indicating that these factors had a minor impact on habitat suitability. The contribution rate (<1.5%) and permutation importance (<5%) of VAPR, SCARD and WIND variables were low, indicating that these variables had limited direct influence on species distribution prediction. Details are shown in Table 2 and Fig 6. Regularization training gain reflects the contribution of variables to model training and avoids overfitting. Test gain reflects the predictive ability of a variable on an independent test set. The AUC demonstrates the overall discrimination accuracy of the model. The connection lies in the joint analysis of the robustness of verifiable variable contributions by the three parties - if a certain variable is significant in training, testing, and AUC, its ecological significance is more reliable; If the results are inconsistent (such as high training gain but low test gain), it may indicate overfitting or variable redundancy.

**Table 2:** Contribution rate of different variables to brucellosis.

Variable	Percent contribution	Permutation importance	Permutation importance SD
POP	69.8	45.1	15.89
NDVI	20.4	30.4	49.83
GDP	4.4	0.6	0.47
VAPR	1.4	4.5	4.72
BIO2	1.2	4.3	11.68
BIO7	0.8	2.6	3.13
BIO11	0.6	7.1	9.38
BIO17	0.5	1.7	1.97
BIO15	0.4	1.7	0.16
SCARD	0.3	1.1	0.85
WIND	0.2	0.9	1.92

**Fig 7:**
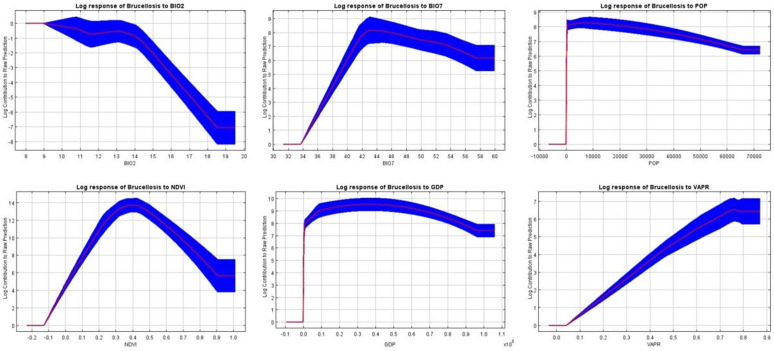
Response curves of some environmental variables in niche model constructed by MaxEnt algorithm.

Fig 7 indicates Population (POP), Gross Domestic Product (GDP), and Mean diurnal temperature range (BIO2) exhibited an inverse relationship with brucellosis incidence, whereby higher levels of POP, GDP, and BIO2 corresponded to decreased incidence risk. Conversely, Normalized Difference Vegetation Index (NDVI) and BIO7 initially increased before declining. The peak incidence risk occurred at an NDVI value of 0.4, and reached its maximum at a BIO7 temperature of 43°C. Additionally, Vapor Pressure (VAPR) initially led to an increase in incidence risk followed by stabilization as it continued to rise. As the BIO2 value increased progressively from 8 to 20, the logarithmic contribution of brucellosis incidence showed a consistent downward trend.

### Risk area prediction

**Fig 8:**
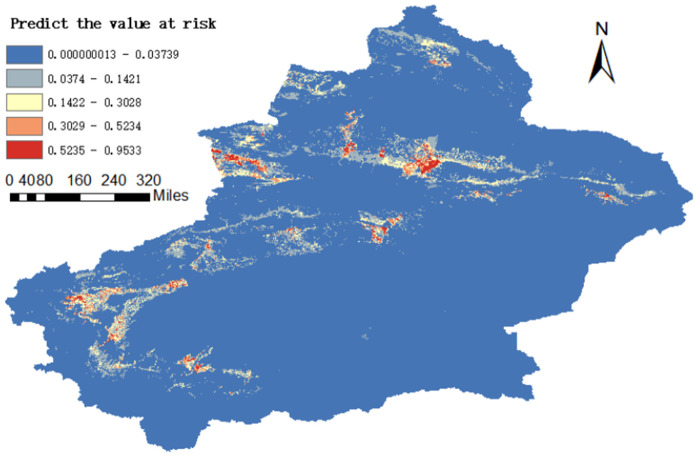
Distribution map of brucellosis transmission risk predicted by MaxEnt niche model in Xinjiang Uygur autonomous region from 2015 to 2023. The map was created in ArcGIS 10.2 software (ESRI Inc., Redlands, CA, USA). The source of the base layer shapefile was from the open access platform: National Platform for Common Geospatial Information Services (www.tianditu.gov.cn).

Fig 8 shows the prediction results of maximum entropy model. The high-risk areas are mainly concentrated in the central and western regions, Urumqi City, Changji City and Yining City in the middle are high-risk areas, Kashgar Prefecture in the southwest is also a high-incidence area of brucellosis; other areas are scattered. By comparing the predicted risk area with the distribution of reported cases, it can be seen that most cases fall within the risk area, and the prediction result is good. However, because the cases were only located at the county level, the location of some sporadic cases was not accurate enough.

## Discussion

In recent years, the incidence of brucellosis in Xinjiang has shown a rebound trend. Since 2021, the number of cases has been increasing annually, accompanied by a gradual rise in the average age of infection onset. The epidemic peaks between May and August, which aligns with previous research findings [26]. In spring, as temperatures rise, bacteria become more active, facilitating the spread of pathogens through the secretions of infected animals. Summer’s high temperatures and humidity provide favorable conditions for bacterial proliferation. Additionally, the frequent breeding and growth activities of animals further increase the risk of transmission [27].

Extensive research has confirmed that climate change significantly influences the population biology characteristics of zoonotic hosts [28]. Climate plays a pivotal role in the zoonotic origins, pathogenic mechanisms, and transmission pathways of infectious diseases. Previous studies have successfully employed the MaxEnt model to analyze several zoonotic diseases, including dengue fever, hemorrhagic fever, and plague [29]. The GAM model has also demonstrated its robust ability to capture climate factors and has been widely applied in vector-borne disease research [30,31]. This study leverages the GAM and MaxEnt models to explore the relationship between human brucellosis and environmental factors, as well as their lagged effects.

The GAM model findings indicate that high temperatures, high precipitation, high wind speed, and low relative humidity are risk factors for brucellosis, exhibiting specific lagged and cumulative effects. This may be attributed to the natural environment being conducive to the survival of Brucella. Increased average temperatures and wind speeds heighten the risk of infection. Brucellosis shows marked seasonality, with higher prevalence in spring and summer. Microbiological studies have shown that 37°C is the optimal growth temperature for Brucella. As a zoonotic pathogen closely tied to animal husbandry, Brucella exhibits higher reproductive rates and biological activity in warm seasons compared to cold seasons. In spring and summer, declining feeding and weight gain rates in cattle and sheep weaken their immunity, increasing the risk of brucellosis infection [32]. Rising temperatures promote the development and reproduction of Brucella within hosts and increase exposure opportunities for susceptible animals and humans [33]. Specifically, the high temperatures in late spring and early summer intensify livestock farming activities such as shearing, breeding, meat processing, and commercialization, thereby increasing the risk of susceptible animals coming into contact with contaminated animal products [34,35]. In contrast, low winter temperatures may suppress pathogen activity, reducing the risk of brucellosis transmission. When monthly precipitation is below 20 mm, the risk increases with rising precipitation; after reaching 20 mm, the risk stabilizes. This finding differs from those of other studies [36]. In Xinjiang’s arid and semi-arid climate zones (annual precipitation <150 mm), increased precipitation significantly enhances pasture growth, prompting herders to expand grazing ranges and increasing human-animal contact frequency. Precipitation forms temporary water bodies that serve as transmission media for Brucella. Experiments have shown that in a 15–20°C precipitation environment, the pathogen’s survival time extends to 90 days, and post-rainfall surface runoff accelerates pathogen dispersal. Soil samples from pastures have shown a 2.1-fold increase in Brucella positivity. Higher relative humidity reduces infection risk. Multiple studies have confirmed that relative humidity affects the survival ability of Brucella. Bacterial culture experiments indicate that the bacterial count decreases with rising relative humidity, suggesting that high relative humidity is unfavorable for Brucella survival. Furthermore, suitable relative humidity significantly impacts host estrus, which is crucial for the transmission of human brucellosis among susceptible livestock. This implies that regulating relative humidity in endemic areas can influence Brucella pathogen activity, thereby aiding in the prevention and control of transmission. Xinjiang’s unique geographical location leads to frequent grazing activities in late spring. Suitable temperatures not only provide a favorable environment for Brucella but also create ideal conditions for host-animal reproduction, increasing the risk of human contact with infected animals [37,38]. Meanwhile, due to insufficient awareness of brucellosis prevention and treatment, delays in diagnosis and treatment prolong case-reporting times [39].

The MaxEnt model results indicate that population density (POP) has the highest contribution rate, consistent with other domestic studies. The incidence of NDVI and brucellosis exhibits a trend characterized by an initial increase followed by a subsequent decline. This pattern may be attributed to the fact that moderate vegetation coverage offers suitable habitats and sufficient food resources for host animals, thereby facilitating the transmission of pathogens. In contrast, excessive vegetation coverage may limit human exposure to hosts or enhance the dilution effect within the ecosystem, ultimately reducing disease risk. A similar relationship between BIO7 and the incidence rate suggests that moderate temperature fluctuations may be conducive to the survival and dissemination of Brucella. However, extreme temperature variations may impair host immune responses or exceed the physiological tolerance of the pathogen, resulting in a decline in the incidence rate. This phenomenon highlights that the impact of environmental factors on brucellosis transmission is non-linear and operates within an optimal range. Beyond this threshold, ecological or climatic conditions may act as limiting factors, thereby suppressing the spread of the disease. One study found that NDVI with a 6-month lag was significantly positively correlated with IHB (*β* = 0.349, *P* = 0.036), confirming that NDVI significantly influences brucellosis transmission through similar seasonal fluctuations [40]. Over 65% of Inner Mongolia Autonomous Region is covered by grassland pastures, where vegetation coverage periodically increases from January to August. High vegetation coverage during grazing seasons not only increases exposure to Brucella-carrying hosts but also leads to frequent animal activity, continuously contaminating the environment. Susceptible animals ingesting contaminated vegetation face an elevated infection risk. Moreover, the favorable hydrothermal conditions created by vegetation growth prolong Brucella survival in water and soil (up to 120 days). GDP is the second most significant environmental factor, showing a negative correlation with brucellosis in Xinjiang. Economic development is inversely related to brucellosis incidence, suggesting that higher economic levels may lead to greater public health awareness and improved disease prevention measures. A study on the spatiotemporal trends of human brucellosis in China based on interpretability analysis found that its transmission is positively correlated with GDP and negatively correlated with humidity. Overcrowded livestock increases direct transmission opportunities among animals, while higher GDP implies better sanitary conditions and medical accessibility, thereby improving disease detection rates. Socioeconomic drivers may play a key role in the transmission of human brucellosis. The growing demand for animal protein leads to increased livestock production, slaughter, and transportation activities, elevating human exposure to animal hosts and their vectors. The results suggest a potential negative correlation between BIO2 and brucellosis, i.e., rising BIO2 levels may suppress certain factors associated with brucellosis, reducing its logarithmic contribution value. The relationship between BIO7 and brucellosis exhibits a complex nonlinear association, where increases within a certain range promote a rise in the logarithmic contribution value, but beyond a specific threshold, the effect weakens. This may imply an optimal threshold for BIO7’s impact on brucellosis. Meanwhile, VAPR shows a positive correlation with the logarithmic contribution value of brucellosis, indicating that increases in VAPR continuously elevate brucellosis-related indicators. However, at present, only associations can be observed, and causality cannot be determined. Further research is needed to clarify the specific mechanisms by which these factors influence brucellosis, providing more precise evidence for disease prevention and control.

Furthermore, climatic factors play a crucial role in either increasing or reducing human contact with infected animals. Studies have found that higher environmental temperatures, combined with lower relative humidity and wind speed, are associated with heightened brucellosis activity [41]. These findings hold particular significance in addressing the challenges of climate change, as it may further alter these environmental conditions, potentially exacerbating brucellosis outbreak risks. Understanding these dynamics is essential for local health departments and provides a scientific basis for formulating targeted prevention strategies [42,43].

## Supporting information

S1 Data Collinearity test. (CSV)

S2 Data MaxEnt results. (CSV)
